# Finding voice

**DOI:** 10.1093/oncolo/oyaf289

**Published:** 2025-10-13

**Authors:** Grace S Kao

**Affiliations:** Department of Pain Medicine, The University of Texas MD Anderson Cancer Center, Houston, TX, 77030, USA

**Keywords:** Cancer survivorship, pain psychology, voice loss, identity, meaning making

## Abstract

This narrative essay explores the experience of a cancer survivor confronting progressive voice loss after head and neck cancer treatment. Through his evolving relationship with speech, identity, and connection, both patient and psychologist reflect on the meaning and presence of voice, spoken and otherwise, as a tool for healing, expression, and connection.

Implications for Practice The piece highlights the importance of attending to identity shifts in long-term cancer survivors, especially as they cope with functional losses like speech. It also illustrates how expressive writing, relational depth, and clinician self-reflection can support meaning-making and adaptation in survivorship care. Clinicians are reminded that healing often emerges through mutual vulnerability and relational presence.

Mr M’s voice had always found its rhythm in the gym. Amid the clatter of weights and the steady cadence of training sets, he coached with quiet authority, his soft voice an anchoring presence for his many student athletes.

These students and his role as a youth weightlifting coach were among the first parts of life Mr M shared with me. When we met, he was 12 years out from chemoradiotherapy for squamous cell carcinoma of the tonsillar fossa. The cancer was long treated, but its legacy lingered through chronic pain and a myriad of other symptoms. He returned regularly to the cancer center, where I practice as a health psychologist. There, his multidisciplinary care included pain psychology, where our work began.

Mr M was sturdily built and soft-spoken, a man in his 60s who, in our first session, offered simply, “I don’t talk much.” Cancer treatment had damaged the muscles and nerves essential for speech. But even before illness, he shared, he’d “always been quiet.” Cancer had forced a disruptive reckoning with dependence and vulnerability. In early sessions, we explored the discomfort of that shift from identifying with stoic silence to appreciating a closer connection.

*Silence, I understood. As a child, I was described as painfully shy. When my family immigrated when I was four, the unfamiliarity was daunting. “She doesn’t talk much, does she?” other parents would say. Spoken words revealed a private world I wasn’t always ready to share. Working with Mr M stirred reflections on how we depend on voice, fear its loss and misuse, and how deep connection often requires enough courage to be heard.*

As our sessions deepened, so did Mr M’s willingness to share. His words came slowly but began to carry more weight. Over time, his pain narrative revealed a largely buried ache: the death of his son many years earlier. His sorrow had gone unexplored and, in many instances, unnamed. I introduced expressive writing as another therapeutic tool, and he wrote—not just about grief, but of joyful memories, forgiveness, and love in the face of pain. As he wrote, the story of his son’s life shifted from its heartbreaking final moments to one marked more fully by connection. In writing, Mr M found another kind of voice, and through that voice, a path toward healing.

Several months into our work, Mr M’s physical voice began to falter. The voice he had wielded with such confidence began to waver without warning. Mr M described increasingly frequent episodes of throat tightening and stilted words. Coaching, once a source of joy and clarity, became straining and disorienting.

*I think often about that irony—that someone who “doesn’t talk much” could be so profoundly defined by voice. As Mr M struggled with its loss, I found myself remembering moments of unwilling silence. Throughout childhood and beyond, silence had been both a shield and survival strategy, one I frequently defaulted to, to avoid shame or error. Even now, as a psychologist whose work depends on language, there are moments when I feel a sobering responsibility in using my voice. Through my work with Mr M, I became more attuned to how to find balance between caution in quietness and developing a voice that brings thoughtful impact.*

As Mr M’s voice loss progressed, our sessions turned toward identity: grieving the “old self” while stepping into the “new.” This new self, he acknowledged, might lose his natural voice but not his ability to connect. At the gym, Mr M shared his condition with his students and invited experienced lifters to mentor others. At home, he and his wife spoke with more thoughtfulness and intention. He began to empower others to speak and lead. Even without words, he continued to convey care.

*Watching Mr M reorient around what endured—values, relationships, presence—was deeply moving. Together, we decided to document this part of his story in writing. He spoke. I wrote. For both of us, words, once often withheld, were gifts in this process, no longer taken for granted, but shared with more intention and grace.*

A month ago, Mr M reached out to schedule an ad hoc session. He had learned he would need a total laryngectomy due to progressive vocal cord paralysis. When we met, he said he was ready, or trying to be. He had begun recording coaching videos for his students and leaving voicemails for loved ones who wanted to remember his natural voice. He was preparing to accept all that would change. Still, what he seemed to mourn most wasn’t the sound of his voice itself, but the care and support that voice had helped him offer.

I validated his fears. We honored his growth. I reminded him that his voice, literal or not, would continue resonating in those he had cared for. We spoke of yet another version of self, still unfolding.

*I chose my words carefully.* “How are you coping, with all of this? With knowing your voice will not be the same?” I asked.

He paused. “I don’t talk much anyway,” he said, echoing words from our first meeting.

I sat with the weight of that full-circle moment.

“Aloud or not,” I replied, “You’ve expressed—and given—so much.”

## Data sharing plans

This submission is a narrative essay and does not include original research data, documentation, or code. Therefore, no data sharing is applicable.

